# *catena*-Poly[[aqua­(4-ethyl­benzoic acid-κ*O*)lanthanum(III)]-tri-μ-4-ethyl­benzoato]

**DOI:** 10.1107/S160053681000190X

**Published:** 2010-01-20

**Authors:** Juan Yang, Jiantong Li, Qiufen Wang

**Affiliations:** a Department of Physical Chemistry, Henan Polytechnic University, Jiaozuo, 454003, People’s Republic of China

## Abstract

The reaction of lanthanum nitrate and 4-ethyl­benzoic acid (EBAH) in aqueous solution yielded the title polymer, [La(C_9_H_9_O_2_)_3_(C_9_H_10_O_2_)(H_2_O)]_*n*_. The asymmetric unit contains one La^III^ atom, three 4-ethyl­benzoate (EBA) ligands, one neutral EBAH ligand and one coordinated water mol­ecule. Each La^III^ ion is eight-coordinated by six O atoms from six bridging-bidentate EBA ligands, one O atom from a monodentate EBAH ligand and one water O atom in a distorted bicapped trigonal-prismatic geometry. The adjacent La^III^ ions are linked by the carboxyl­ate groups of EBA ligands in a bridging-bidetate coordination mode, resulting in an infinite chain structure along the *c* axis. O—H⋯O hydrogen-bonding inter­actions involving the water mol­ecules, carboxyl­ate groups and carboxyl H atoms are formed within the one-dimensional polymer. One of the ethyl groups is disordered over two positions with occupancies of 0.717 (7) and 0.283 (7).

## Related literature

For information on lanthanum complexes, see: Ishii *et al.* (2002 ▶); Kim *et al.* (2001 ▶); Luneau & Rey (2005 ▶); Wang *et al.* (2006 ▶); Yu *et al.* (2003 ▶).
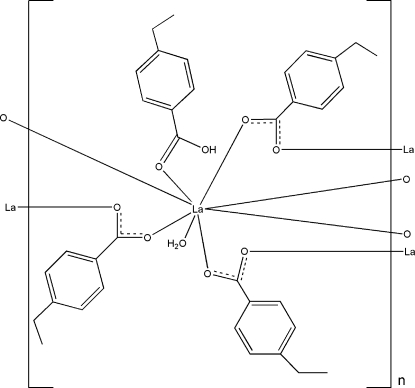


## Experimental

### 

#### Crystal data

[La(C_9_H_9_O_2_)_3_(C_9_H_10_O_2_)(H_2_O)]
*M* *_r_* = 754.58
Triclinic, 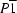

*a* = 9.5319 (3) Å
*b* = 14.0378 (5) Å
*c* = 14.9847 (5) Å
α = 65.024 (2)°
β = 74.942 (2)°
γ = 74.734 (2)°
*V* = 1727.91 (10) Å^3^
*Z* = 2
Mo *K*α radiation
μ = 1.29 mm^−1^
*T* = 296 K
0.35 × 0.32 × 0.23 mm

#### Data collection

Bruker APEXII CCD diffractometer
Absorption correction: multi-scan (*SADABS*; Bruker, 2007 ▶) *T*_min_ = 0.643, *T*_max_ = 0.744
22559 measured reflections
7733 independent reflections
6206 reflections with *I* > 2σ(*I*)
*R*_int_ = 0.050

#### Refinement

*R*[*F* ^2^ > 2σ(*F* ^2^)] = 0.038
*wR*(*F* ^2^) = 0.077
*S* = 1.01
7733 reflections
427 parameters
14 restraints
H-atom parameters constrained
Δρ_max_ = 0.63 e Å^−3^
Δρ_min_ = −0.73 e Å^−3^

### 

Data collection: *APEX2* (Bruker, 2007 ▶); cell refinement: *SAINT* (Bruker, 2007 ▶); data reduction: *SAINT*; program(s) used to solve structure: *SHELXS97* (Sheldrick, 2008 ▶); program(s) used to refine structure: *SHELXL97* (Sheldrick, 2008 ▶); molecular graphics: *SHELXTL* (Sheldrick, 2008 ▶); software used to prepare material for publication: *SHELXTL*.

## Supplementary Material

Crystal structure: contains datablocks global, I. DOI: 10.1107/S160053681000190X/gk2253sup1.cif

Structure factors: contains datablocks I. DOI: 10.1107/S160053681000190X/gk2253Isup2.hkl

Additional supplementary materials:  crystallographic information; 3D view; checkCIF report

## Figures and Tables

**Table 1:** Selected bond lengths (Å)

La1—O7^i^	2.446 (2)
La1—O1	2.451 (2)
La1—O2^ii^	2.457 (2)
La1—O6^i^	2.466 (2)
La1—O8	2.479 (2)
La1—O5	2.581 (2)
La1—O9	2.624 (2)
La1—O3	2.672 (2)

Symmetry codes: (i) 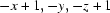; (ii) 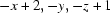.

**Table 2:** Hydrogen-bond geometry (Å, °)

*D*—H⋯*A*	*D*—H	H⋯*A*	*D*⋯*A*	*D*—H⋯*A*
O4—H4*A*⋯O5	0.82	1.84	2.652 (3)	171
O9—H9*B*⋯O2	0.82	2.04	2.829 (3)	161

**Fig. 1.**
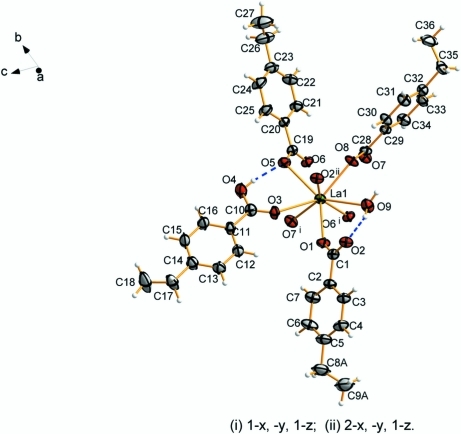
The coordination environment of LaIII in the title complex with the atom-labeling scheme[symmetry codes: (i) -x + 1, -y, -z + 1; (ii) -x + 2, -y, -z + 1] Displacement ellipsoids for non-hydrogen atoms are drawn at the 30% probability level. Hydrogen bonds are shown as dashed lines.

**Fig. 2.**
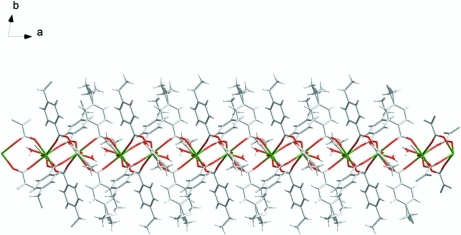
The one-dimensional chain structure of the title compound.

## supplementary crystallographic information

### Comment

Recently, the use of lanthanide salts for the synthesis of coordination polymer has attracted more and more attentions due to their high coordination numbers along with distinguished magnetic and luminescent properties (Ishii *et al.*, 2002; Luneau & Rey, 2005; Yu *et al.*, 2003). As an important family of multidentate O-donor ligands, aromatic carboxylate ligands have been extensively employed in the preparation of metal-organic complexes because of their potential properties and intriguing structural topologies (Kim *et al.*, 2001; Wang *et al.*, 2006). Herein, we report the structure of the title La^III^ coordibation polymer.

The asymmetric unit of the title compound, [La(C_9_H_9_O_2_)_3_(C_9_H_10_O_2_)(H_2_O)]_n_ contains one La^III^ cation, three anionic EBA ligands, one neutral ligand EBAH and one water molecule, as illustrated in Fig. 1. The La^III^ atom is coordinated by eight O atoms from six bridging-bidentate EBA ligands, one monodentate EBAH ligand and one water molecule. The La^III^ center adopts a distorted bicapped trigonal prism geometry. The carboxylate groups of EBA ligands link the adjacent La^III^ ions in bridging-bidetate coordination modeto form an infinite chain structure running along the c axis. The shortest intermetallic distance La···La is 4.2601 (4) Å, indicating a weak metal-metal interaction. The coordinating water, carboxylate O atoms of EBA ligands and carboxylic H atom of EBAH ligand are involved in O—H···O hydrogen-bonding interactions. These hydrogen bonds are all intramolecular, *i.e.* stabilize the one-dimensional chain structure of the title polymer (Table 2).

### Experimental

A mixture of La(NO_3_)_3_.6H_2_O (0.225 g, 0.52 mmol), EBAH (0.126 g, 0.84 mmol), melamine (0.026 g, 0.20 mmol) and distilled water (10 ml) was sealed in a 25 ml Teflon-lined stainless autoclave. The mixture was heated at 423 K for 7 days to give the colorless prism crystals suitable for X-ray diffraction analysis.

### Refinement

All H atoms bounded to C atoms were positioned geometrically and allowed to ride on their parent atoms, with C (phenyl)—H = 0.93 Å, C (methyl)—H = 0.96 Å, and C (methylene)—H = 0.97 Å, respectively with *U*_iso_ (H) = 1.2 *U*_eq_(C). The ethyl group C8—C9 was treated as disordered over two sites, with refined ccupancies 0.717 (7) and 0.283 (7). Positions of the water H atoms and the carboxylic H atom were found from a difference Fourier map and O—H distances constrained to 0.82 Å. Among 14 restraints used in the refinement are those used to restrain geometry of the disordered ethyl group and SHELXL-97 ISOR restraint imposed on the displacement ellipsoids of C27 and C9A.

### Crystal data

**Table:**

[La(C_9_H_9_O_2_)_3_(C_9_H_10_O_2_)(H_2_O)]	*Z* = 2
*M**_r_* = 754.58	*F*(000) = 768
Triclinic, *P*1	*D*_x_ = 1.450 Mg m^−^^3^
Hall symbol: -P 1	Mo *K*α radiation, λ = 0.71073 Å
*a* = 9.5319 (3) Å	Cell parameters from 4271 reflections
*b* = 14.0378 (5) Å	θ = 1.6–27.4°
*c* = 14.9847 (5) Å	µ = 1.29 mm^−^^1^
α = 65.024 (2)°	*T* = 296 K
β = 74.942 (2)°	Prism, colorless
γ = 74.734 (2)°	0.35 × 0.32 × 0.23 mm
*V* = 1727.91 (10) Å^3^	

### Data collection

**Table:**

Bruker APEXII CCD diffractometer	7733 independent reflections
Radiation source: fine-focus sealed tube	6206 reflections with *I* > 2σ(*I*)
graphite	*R*_int_ = 0.050
φ and ω scans	θ_max_ = 27.4°, θ_min_ = 1.6°
Absorption correction: multi-scan (*SADABS*; Bruker, 2007)	*h* = −12→12
*T*_min_ = 0.643, *T*_max_ = 0.744	*k* = −17→18
22559 measured reflections	*l* = −19→19

### Refinement

**Table:**

Refinement on *F*^2^	Secondary atom site location: difference Fourier map
Least-squares matrix: full	Hydrogen site location: inferred from neighbouring sites
*R*[*F*^2^ > 2σ(*F*^2^)] = 0.038	H-atom parameters constrained
*wR*(*F*^2^) = 0.077	*w* = 1/[σ^2^(*F*_o_^2^) + (0.028*P*)^2^ + 0.4*P*] where *P* = (*F*_o_^2^ + 2*F*_c_^2^)/3
*S* = 1.01	(Δ/σ)_max_ = 0.001
7733 reflections	Δρ_max_ = 0.63 e Å^−^^3^
427 parameters	Δρ_min_ = −0.73 e Å^−^^3^
14 restraints	Extinction correction: *SHELXL97* (Sheldrick, 2008)
Primary atom site location: structure-invariant direct methods	Extinction coefficient: none

### Fractional atomic coordinates and isotropic or equivalent isotropic displacement parameters (Å^2^)

**Table:**

	*x*	*y*	*z*	*U*_iso_*/*U*_eq_	Occ. (<1)
La1	0.70393 (2)	0.005772 (14)	0.529430 (14)	0.03202 (7)	
O1	0.8939 (2)	−0.14429 (17)	0.60351 (17)	0.0452 (6)	
O2	1.1201 (2)	−0.12633 (17)	0.51839 (17)	0.0425 (6)	
O3	0.7662 (3)	0.01235 (19)	0.69039 (17)	0.0476 (6)	
O4	0.5733 (3)	0.1064 (2)	0.7549 (2)	0.0716 (9)	
H4A	0.5546	0.1300	0.6982	0.086*	
O5	0.5373 (2)	0.16617 (17)	0.56793 (17)	0.0438 (6)	
O6	0.3932 (3)	0.11669 (17)	0.50875 (17)	0.0451 (6)	
O7	0.4662 (3)	0.08426 (18)	0.32269 (17)	0.0480 (6)	
O8	0.6584 (3)	0.11814 (19)	0.35678 (17)	0.0483 (6)	
O9	0.9081 (3)	−0.0388 (2)	0.39017 (18)	0.0582 (7)	
H9A	0.9185	0.0072	0.3337	0.087*	
H9B	0.9818	−0.0684	0.4164	0.087*	
C1	1.0302 (4)	−0.1780 (2)	0.5892 (2)	0.0369 (8)	
C2	1.0876 (4)	−0.2861 (2)	0.6599 (2)	0.0382 (8)	
C3	1.2323 (4)	−0.3347 (3)	0.6403 (3)	0.0510 (10)	
H3	1.2948	−0.2994	0.5832	0.061*	
C4	1.2843 (5)	−0.4349 (3)	0.7050 (3)	0.0686 (12)	
H4	1.3809	−0.4675	0.6900	0.082*	
C5	1.1953 (6)	−0.4879 (3)	0.7917 (3)	0.0717 (13)	
C6	1.0516 (5)	−0.4374 (3)	0.8123 (3)	0.0695 (13)	
H6	0.9906	−0.4712	0.8711	0.083*	
C7	0.9980 (4)	−0.3380 (3)	0.7470 (3)	0.0545 (10)	
H7	0.9011	−0.3058	0.7615	0.065*	
C8A	1.243 (2)	−0.6017 (18)	0.8639 (16)	0.110 (7)	0.717 (7)
H8A	1.2931	−0.5979	0.9108	0.131*	0.717 (7)
H8B	1.1553	−0.6318	0.9019	0.131*	0.717 (7)
C9A	1.3372 (10)	−0.6726 (6)	0.8198 (6)	0.119 (3)	0.717 (7)
H9C	1.3514	−0.7432	0.8701	0.178*	0.717 (7)
H9D	1.4307	−0.6496	0.7902	0.178*	0.717 (7)
H9E	1.2930	−0.6729	0.7693	0.178*	0.717 (7)
C8B	1.271 (7)	−0.592 (5)	0.863 (4)	0.110 (7)	0.283 (7)
H8C	1.2841	−0.6473	0.8382	0.131*	0.283 (7)
H8D	1.3690	−0.5825	0.8627	0.131*	0.283 (7)
C9B	1.199 (3)	−0.6280 (15)	0.9635 (18)	0.119 (3)	0.283 (7)
H9F	1.2268	−0.7040	0.9943	0.178*	0.283 (7)
H9G	1.0938	−0.6101	0.9652	0.178*	0.283 (7)
H9H	1.2256	−0.5945	0.9993	0.178*	0.283 (7)
C10	0.6986 (4)	0.0379 (3)	0.7597 (3)	0.0446 (8)	
C11	0.7491 (4)	−0.0046 (3)	0.8568 (3)	0.0461 (9)	
C12	0.8705 (5)	−0.0842 (3)	0.8747 (3)	0.0613 (11)	
H12	0.9191	−0.1116	0.8259	0.074*	
C13	0.9214 (5)	−0.1240 (3)	0.9641 (3)	0.0703 (13)	
H13	1.0038	−0.1779	0.9743	0.084*	
C14	0.8540 (5)	−0.0862 (3)	1.0379 (3)	0.0637 (11)	
C15	0.7330 (5)	−0.0069 (4)	1.0200 (3)	0.0802 (15)	
H15	0.6844	0.0199	1.0691	0.096*	
C16	0.6812 (5)	0.0343 (4)	0.9305 (3)	0.0711 (13)	
H16	0.5995	0.0888	0.9202	0.085*	
C17	0.9168 (6)	−0.1315 (4)	1.1346 (3)	0.0830 (15)	
H17A	0.9530	−0.2072	1.1509	0.100*	
H17B	1.0009	−0.0983	1.1223	0.100*	
C18	0.8160 (7)	−0.1179 (5)	1.2215 (4)	0.124 (2)	
H18A	0.7852	−0.0431	1.2089	0.186*	
H18B	0.8651	−0.1520	1.2785	0.186*	
H18C	0.7312	−0.1494	1.2344	0.186*	
C19	0.4269 (3)	0.1873 (2)	0.5257 (2)	0.0353 (7)	
C20	0.3432 (3)	0.2980 (2)	0.4932 (2)	0.0368 (8)	
C21	0.2418 (4)	0.3307 (3)	0.4299 (3)	0.0502 (9)	
H21	0.2218	0.2815	0.4105	0.060*	
C22	0.1699 (5)	0.4353 (3)	0.3953 (3)	0.0617 (11)	
H22	0.1025	0.4553	0.3527	0.074*	
C23	0.1952 (5)	0.5096 (3)	0.4220 (3)	0.0616 (11)	
C24	0.2970 (5)	0.4781 (3)	0.4853 (4)	0.0733 (14)	
H24	0.3158	0.5278	0.5045	0.088*	
C25	0.3715 (5)	0.3726 (3)	0.5205 (3)	0.0603 (11)	
H25	0.4399	0.3528	0.5623	0.072*	
C26	0.1122 (6)	0.6239 (3)	0.3830 (4)	0.0977 (18)	
H26A	0.0694	0.6333	0.3271	0.117*	
H26B	0.0312	0.6329	0.4351	0.117*	
C27	0.1952 (8)	0.7071 (4)	0.3512 (5)	0.133 (2)	
H27A	0.1389	0.7739	0.3146	0.199*	
H27B	0.2859	0.6923	0.3090	0.199*	
H27C	0.2168	0.7111	0.4085	0.199*	
C28	0.5754 (4)	0.1295 (2)	0.2983 (2)	0.0381 (8)	
C29	0.6127 (3)	0.2007 (2)	0.1914 (2)	0.0367 (8)	
C30	0.7214 (4)	0.2619 (3)	0.1596 (3)	0.0476 (9)	
H30	0.7741	0.2584	0.2055	0.057*	
C31	0.7529 (4)	0.3279 (3)	0.0608 (3)	0.0527 (10)	
H31	0.8253	0.3694	0.0415	0.063*	
C32	0.6798 (4)	0.3339 (3)	−0.0099 (3)	0.0513 (10)	
C33	0.5723 (5)	0.2719 (3)	0.0215 (3)	0.0579 (11)	
H33	0.5222	0.2739	−0.0251	0.069*	
C34	0.5373 (4)	0.2069 (3)	0.1209 (3)	0.0520 (10)	
H34	0.4628	0.1670	0.1405	0.062*	
C35	0.7126 (5)	0.4099 (3)	−0.1179 (3)	0.0732 (13)	
H35A	0.8179	0.4101	−0.1368	0.088*	
H35B	0.6846	0.3844	−0.1606	0.088*	
C36	0.6326 (7)	0.5210 (4)	−0.1346 (4)	0.117 (2)	
H36A	0.5286	0.5209	−0.1135	0.176*	
H36B	0.6519	0.5650	−0.2043	0.176*	
H36C	0.6659	0.5488	−0.0967	0.176*	

### Atomic displacement parameters (Å^2^)

**Table:**

	*U*^11^	*U*^22^	*U*^33^	*U*^12^	*U*^13^	*U*^23^
La1	0.02659 (10)	0.03400 (11)	0.03639 (11)	−0.00694 (7)	−0.01086 (7)	−0.01010 (8)
O1	0.0330 (13)	0.0440 (13)	0.0504 (15)	−0.0023 (11)	−0.0126 (11)	−0.0096 (11)
O2	0.0350 (13)	0.0397 (12)	0.0501 (14)	−0.0112 (10)	−0.0132 (11)	−0.0082 (11)
O3	0.0458 (14)	0.0621 (16)	0.0420 (14)	−0.0068 (12)	−0.0105 (12)	−0.0263 (12)
O4	0.0567 (18)	0.100 (2)	0.0626 (18)	0.0178 (16)	−0.0241 (15)	−0.0452 (17)
O5	0.0373 (13)	0.0446 (13)	0.0541 (15)	−0.0007 (11)	−0.0208 (11)	−0.0193 (12)
O6	0.0475 (14)	0.0380 (13)	0.0555 (15)	−0.0107 (11)	−0.0110 (12)	−0.0201 (11)
O7	0.0421 (14)	0.0552 (15)	0.0432 (14)	−0.0194 (12)	−0.0068 (11)	−0.0092 (12)
O8	0.0434 (14)	0.0552 (15)	0.0399 (14)	−0.0118 (12)	−0.0186 (11)	−0.0033 (12)
O9	0.0387 (14)	0.0818 (19)	0.0522 (16)	−0.0015 (13)	−0.0134 (12)	−0.0255 (14)
C1	0.0369 (19)	0.0352 (17)	0.044 (2)	−0.0107 (15)	−0.0145 (16)	−0.0136 (15)
C2	0.0368 (19)	0.0337 (17)	0.046 (2)	−0.0073 (14)	−0.0195 (16)	−0.0090 (15)
C3	0.048 (2)	0.044 (2)	0.056 (2)	−0.0015 (17)	−0.0182 (19)	−0.0126 (18)
C4	0.063 (3)	0.052 (2)	0.079 (3)	0.010 (2)	−0.026 (2)	−0.018 (2)
C5	0.084 (3)	0.045 (2)	0.073 (3)	−0.002 (2)	−0.037 (3)	−0.002 (2)
C6	0.072 (3)	0.055 (2)	0.062 (3)	−0.023 (2)	−0.019 (2)	0.008 (2)
C7	0.046 (2)	0.052 (2)	0.060 (3)	−0.0148 (18)	−0.0183 (19)	−0.0066 (19)
C8A	0.118 (10)	0.072 (6)	0.097 (5)	0.014 (8)	−0.043 (7)	0.003 (4)
C9A	0.141 (6)	0.069 (4)	0.135 (6)	0.008 (4)	−0.062 (5)	−0.022 (4)
C8B	0.118 (10)	0.072 (6)	0.097 (5)	0.014 (8)	−0.043 (7)	0.003 (4)
C9B	0.141 (6)	0.069 (4)	0.135 (6)	0.008 (4)	−0.062 (5)	−0.022 (4)
C10	0.039 (2)	0.052 (2)	0.049 (2)	−0.0111 (17)	−0.0124 (17)	−0.0205 (18)
C11	0.041 (2)	0.058 (2)	0.043 (2)	−0.0056 (17)	−0.0096 (17)	−0.0236 (18)
C12	0.066 (3)	0.067 (3)	0.047 (2)	0.003 (2)	−0.012 (2)	−0.026 (2)
C13	0.071 (3)	0.074 (3)	0.057 (3)	0.011 (2)	−0.021 (2)	−0.024 (2)
C14	0.063 (3)	0.077 (3)	0.049 (2)	−0.005 (2)	−0.016 (2)	−0.022 (2)
C15	0.081 (3)	0.109 (4)	0.052 (3)	0.017 (3)	−0.019 (2)	−0.048 (3)
C16	0.064 (3)	0.088 (3)	0.058 (3)	0.020 (2)	−0.021 (2)	−0.038 (2)
C17	0.089 (4)	0.104 (4)	0.052 (3)	−0.010 (3)	−0.031 (3)	−0.019 (3)
C18	0.109 (5)	0.202 (7)	0.064 (4)	−0.002 (5)	−0.031 (3)	−0.058 (4)
C19	0.0304 (17)	0.0358 (17)	0.0388 (19)	−0.0061 (14)	−0.0045 (14)	−0.0139 (15)
C20	0.0323 (17)	0.0371 (17)	0.0411 (19)	−0.0046 (14)	−0.0063 (15)	−0.0159 (15)
C21	0.051 (2)	0.044 (2)	0.055 (2)	−0.0051 (17)	−0.0187 (19)	−0.0139 (18)
C22	0.058 (3)	0.050 (2)	0.066 (3)	−0.002 (2)	−0.024 (2)	−0.008 (2)
C23	0.054 (3)	0.044 (2)	0.069 (3)	0.0001 (19)	−0.004 (2)	−0.014 (2)
C24	0.087 (3)	0.049 (3)	0.100 (4)	−0.007 (2)	−0.020 (3)	−0.043 (3)
C25	0.066 (3)	0.049 (2)	0.081 (3)	0.001 (2)	−0.028 (2)	−0.037 (2)
C26	0.083 (4)	0.046 (3)	0.128 (5)	0.002 (2)	−0.004 (3)	−0.015 (3)
C27	0.157 (5)	0.058 (3)	0.184 (6)	−0.007 (3)	−0.073 (5)	−0.028 (4)
C28	0.0319 (18)	0.0368 (17)	0.0400 (19)	−0.0034 (15)	−0.0093 (15)	−0.0091 (15)
C29	0.0316 (17)	0.0368 (17)	0.0379 (18)	−0.0031 (14)	−0.0080 (14)	−0.0111 (15)
C30	0.045 (2)	0.056 (2)	0.042 (2)	−0.0144 (18)	−0.0121 (17)	−0.0128 (17)
C31	0.045 (2)	0.058 (2)	0.050 (2)	−0.0221 (18)	0.0001 (18)	−0.0124 (19)
C32	0.058 (2)	0.047 (2)	0.041 (2)	−0.0027 (19)	−0.0059 (19)	−0.0143 (17)
C33	0.077 (3)	0.059 (2)	0.041 (2)	−0.011 (2)	−0.027 (2)	−0.0126 (19)
C34	0.058 (2)	0.051 (2)	0.051 (2)	−0.0212 (19)	−0.0182 (19)	−0.0104 (18)
C35	0.083 (3)	0.076 (3)	0.037 (2)	−0.011 (3)	0.004 (2)	−0.009 (2)
C36	0.133 (5)	0.071 (3)	0.064 (3)	0.011 (3)	0.019 (3)	0.015 (3)

### Geometric parameters (Å, °)

**Table:**

La1—O7^i^	2.446 (2)	C12—C13	1.380 (5)
La1—O1	2.451 (2)	C12—H12	0.9300
La1—O2^ii^	2.457 (2)	C13—C14	1.365 (5)
La1—O6^i^	2.466 (2)	C13—H13	0.9300
La1—O8	2.479 (2)	C14—C15	1.369 (5)
La1—O5	2.581 (2)	C14—C17	1.528 (6)
La1—O9	2.624 (2)	C15—C16	1.383 (5)
La1—O3	2.672 (2)	C15—H15	0.9300
La1—O6	2.989 (2)	C16—H16	0.9300
O1—C1	1.255 (4)	C17—C18	1.455 (6)
O2—C1	1.262 (4)	C17—H17A	0.9700
O2—La1^ii^	2.457 (2)	C17—H17B	0.9700
O3—C10	1.212 (4)	C18—H18A	0.9600
O4—C10	1.318 (4)	C18—H18B	0.9600
O4—H4A	0.8200	C18—H18C	0.9600
O5—C19	1.272 (4)	C19—C20	1.482 (4)
O6—C19	1.255 (4)	C20—C25	1.381 (5)
O6—La1^i^	2.466 (2)	C20—C21	1.385 (5)
O7—C28	1.255 (4)	C21—C22	1.381 (5)
O7—La1^i^	2.446 (2)	C21—H21	0.9300
O8—C28	1.263 (4)	C22—C23	1.358 (6)
O9—H9A	0.8199	C22—H22	0.9300
O9—H9B	0.8200	C23—C24	1.390 (6)
C1—C2	1.494 (4)	C23—C26	1.524 (5)
C2—C7	1.382 (5)	C24—C25	1.399 (5)
C2—C3	1.385 (5)	C24—H24	0.9300
C3—C4	1.377 (5)	C25—H25	0.9300
C3—H3	0.9300	C26—C27	1.428 (7)
C4—C5	1.380 (6)	C26—H26A	0.9700
C4—H4	0.9300	C26—H26B	0.9700
C5—C6	1.389 (6)	C27—H27A	0.9600
C5—C8B	1.527 (12)	C27—H27B	0.9600
C5—C8A	1.530 (9)	C27—H27C	0.9600
C6—C7	1.377 (5)	C28—C29	1.494 (4)
C6—H6	0.9300	C29—C30	1.380 (5)
C7—H7	0.9300	C29—C34	1.388 (5)
C8A—C9A	1.42 (3)	C30—C31	1.378 (5)
C8A—H8A	0.9700	C30—H30	0.9300
C8A—H8B	0.9700	C31—C32	1.376 (5)
C9A—H9C	0.9600	C31—H31	0.9300
C9A—H9D	0.9600	C32—C33	1.379 (5)
C9A—H9E	0.9600	C32—C35	1.520 (5)
C8B—C9B	1.41 (3)	C33—C34	1.385 (5)
C8B—H8C	0.9700	C33—H33	0.9300
C8B—H8D	0.9700	C34—H34	0.9300
C9B—H9F	0.9600	C35—C36	1.491 (6)
C9B—H9G	0.9600	C35—H35A	0.9700
C9B—H9H	0.9600	C35—H35B	0.9700
C10—C11	1.478 (5)	C36—H36A	0.9600
C11—C16	1.374 (5)	C36—H36B	0.9600
C11—C12	1.374 (5)	C36—H36C	0.9600
			
O7^i^—La1—O1	85.28 (8)	C12—C11—C10	119.7 (3)
O7^i^—La1—O2^ii^	138.39 (8)	C11—C12—C13	120.8 (4)
O1—La1—O2^ii^	88.76 (7)	C11—C12—H12	119.6
O7^i^—La1—O6^i^	72.11 (8)	C13—C12—H12	119.6
O1—La1—O6^i^	88.38 (8)	C14—C13—C12	121.7 (4)
O2^ii^—La1—O6^i^	148.91 (8)	C14—C13—H13	119.1
O7^i^—La1—O8	129.46 (8)	C12—C13—H13	119.1
O1—La1—O8	134.90 (8)	C13—C14—C15	117.3 (4)
O2^ii^—La1—O8	81.26 (8)	C13—C14—C17	119.6 (4)
O6^i^—La1—O8	79.01 (8)	C15—C14—C17	123.1 (4)
O7^i^—La1—O5	78.89 (8)	C14—C15—C16	121.6 (4)
O1—La1—O5	137.55 (8)	C14—C15—H15	119.2
O2^ii^—La1—O5	78.19 (7)	C16—C15—H15	119.2
O6^i^—La1—O5	122.55 (7)	C11—C16—C15	120.7 (4)
O8—La1—O5	83.19 (8)	C11—C16—H16	119.7
O7^i^—La1—O9	138.96 (8)	C15—C16—H16	119.7
O1—La1—O9	69.14 (8)	C18—C17—C14	116.4 (4)
O2^ii^—La1—O9	74.56 (8)	C18—C17—H17A	108.2
O6^i^—La1—O9	75.51 (8)	C14—C17—H17A	108.2
O8—La1—O9	65.79 (8)	C18—C17—H17B	108.2
O5—La1—O9	141.12 (8)	C14—C17—H17B	108.2
O7^i^—La1—O3	70.76 (8)	H17A—C17—H17B	107.3
O1—La1—O3	67.79 (7)	C17—C18—H18A	109.5
O2^ii^—La1—O3	68.89 (8)	C17—C18—H18B	109.5
O6^i^—La1—O3	137.01 (8)	H18A—C18—H18B	109.5
O8—La1—O3	142.87 (8)	C17—C18—H18C	109.5
O5—La1—O3	69.82 (7)	H18A—C18—H18C	109.5
O9—La1—O3	122.91 (7)	H18B—C18—H18C	109.5
O7^i^—La1—O6	69.86 (7)	O6—C19—O5	120.5 (3)
O1—La1—O6	154.19 (7)	O6—C19—C20	120.8 (3)
O2^ii^—La1—O6	114.27 (7)	O5—C19—C20	118.7 (3)
O6^i^—La1—O6	77.73 (7)	C25—C20—C21	118.4 (3)
O8—La1—O6	63.95 (7)	C25—C20—C19	120.4 (3)
O5—La1—O6	45.66 (7)	C21—C20—C19	121.0 (3)
O9—La1—O6	126.33 (7)	C22—C21—C20	120.9 (4)
O3—La1—O6	108.39 (7)	C22—C21—H21	119.5
C1—O1—La1	141.5 (2)	C20—C21—H21	119.5
C1—O2—La1^ii^	144.6 (2)	C23—C22—C21	121.5 (4)
C10—O3—La1	136.8 (2)	C23—C22—H22	119.2
C10—O4—H4A	109.2	C21—C22—H22	119.2
C19—O5—La1	104.12 (19)	C22—C23—C24	118.3 (4)
C19—O6—La1^i^	172.4 (2)	C22—C23—C26	119.8 (5)
C19—O6—La1	85.22 (18)	C24—C23—C26	122.0 (4)
La1^i^—O6—La1	102.27 (7)	C23—C24—C25	120.9 (4)
C28—O7—La1^i^	140.7 (2)	C23—C24—H24	119.5
C28—O8—La1	139.7 (2)	C25—C24—H24	119.5
La1—O9—H9A	119.0	C20—C25—C24	119.9 (4)
La1—O9—H9B	105.1	C20—C25—H25	120.0
H9A—O9—H9B	117.6	C24—C25—H25	120.0
O1—C1—O2	123.7 (3)	C27—C26—C23	116.9 (5)
O1—C1—C2	117.6 (3)	C27—C26—H26A	108.1
O2—C1—C2	118.7 (3)	C23—C26—H26A	108.1
C7—C2—C3	119.0 (3)	C27—C26—H26B	108.1
C7—C2—C1	120.4 (3)	C23—C26—H26B	108.1
C3—C2—C1	120.5 (3)	H26A—C26—H26B	107.3
C4—C3—C2	120.3 (4)	C26—C27—H27A	109.5
C4—C3—H3	119.8	C26—C27—H27B	109.5
C2—C3—H3	119.8	H27A—C27—H27B	109.5
C3—C4—C5	121.1 (4)	C26—C27—H27C	109.5
C3—C4—H4	119.5	H27A—C27—H27C	109.5
C5—C4—H4	119.5	H27B—C27—H27C	109.5
C4—C5—C6	118.2 (4)	O7—C28—O8	125.1 (3)
C4—C5—C8B	115.9 (19)	O7—C28—C29	117.8 (3)
C6—C5—C8B	125.3 (17)	O8—C28—C29	117.1 (3)
C4—C5—C8A	123.8 (8)	C30—C29—C34	118.1 (3)
C6—C5—C8A	117.9 (8)	C30—C29—C28	121.8 (3)
C7—C6—C5	121.0 (4)	C34—C29—C28	120.1 (3)
C7—C6—H6	119.5	C31—C30—C29	120.8 (3)
C5—C6—H6	119.5	C31—C30—H30	119.6
C6—C7—C2	120.3 (4)	C29—C30—H30	119.6
C6—C7—H7	119.9	C32—C31—C30	121.6 (4)
C2—C7—H7	119.9	C32—C31—H31	119.2
C9A—C8A—C5	116.0 (15)	C30—C31—H31	119.2
C9A—C8A—H8A	108.3	C31—C32—C33	117.7 (3)
C5—C8A—H8A	108.3	C31—C32—C35	120.8 (4)
C9A—C8A—H8B	108.3	C33—C32—C35	121.5 (4)
C5—C8A—H8B	108.3	C32—C33—C34	121.4 (4)
H8A—C8A—H8B	107.4	C32—C33—H33	119.3
C9B—C8B—C5	116 (3)	C34—C33—H33	119.3
C9B—C8B—H8C	108.2	C33—C34—C29	120.4 (4)
C5—C8B—H8C	108.2	C33—C34—H34	119.8
C5—C8B—H8D	108.2	C29—C34—H34	119.8
H8C—C8B—H8D	107.4	C36—C35—C32	112.5 (3)
C8B—C9B—H9F	109.5	C36—C35—H35A	109.1
C8B—C9B—H9G	109.5	C32—C35—H35A	109.1
H9F—C9B—H9G	109.5	C36—C35—H35B	109.1
C8B—C9B—H9H	109.5	C32—C35—H35B	109.1
H9F—C9B—H9H	109.5	H35A—C35—H35B	107.8
H9G—C9B—H9H	109.5	C35—C36—H36A	109.5
O3—C10—O4	122.7 (3)	C35—C36—H36B	109.5
O3—C10—C11	123.2 (3)	H36A—C36—H36B	109.5
O4—C10—C11	114.1 (3)	C35—C36—H36C	109.5
C16—C11—C12	117.8 (4)	H36A—C36—H36C	109.5
C16—C11—C10	122.5 (3)	H36B—C36—H36C	109.5

Symmetry codes: (i) −*x*+1, −*y*, −*z*+1; (ii) −*x*+2, −*y*, −*z*+1.

### Hydrogen-bond geometry (Å, °)

**Table:**

*D*—H···*A*	*D*—H	H···*A*	*D*···*A*	*D*—H···*A*
O4—H4A···O5	0.82	1.84	2.652 (3)	171
O9—H9B···O2	0.82	2.04	2.829 (3)	161
